# Aortic Risks Prediction Models after Cardiac Surgeries Using Integrated Data

**DOI:** 10.3390/jpm12040637

**Published:** 2022-04-15

**Authors:** Iuliia Lenivtceva, Dmitri Panfilov, Georgy Kopanitsa, Boris Kozlov

**Affiliations:** 1 National Center for Cognitive Research, ITMO University, 49 Kronverskiy Prospect, 197101 Saint-Petersburg, Russia; georgy.kopanitsa@gmail.com; 2 Cardiology Research Institute, Tomsk National Research Medical Center of the Russian Academy of Science, 634012 Tomsk, Russia; pand2006@yandex.ru (D.P.); bnkozlov@yandex.ru (B.K.); 3 Almazov National Medical Research Center, 2 Akkuratova Street, 197341 Saint-Petersburg, Russia

**Keywords:** postoperative risks, aortic aneurysm, integrated data, predictive modeling, feature extraction, machine learning

## Abstract

The complications of thoracic aortic disease include aortic dissection and aneurysm. The risks are frequently compounded by many cardiovascular comorbidities, which makes the process of clinical decision making complicated. The purpose of this study is to develop risk predictive models for patients after thoracic aneurysm surgeries, using integrated data from different medical institutions. Seven risk features were formulated for prediction. The CatBoost classifier performed best and provided an ROC AUC of 0.94–0.98 and an F-score of 0.95–0.98. The obtained results are widely in line with the current literature. The obtained findings provide additional support for clinical decision making, guiding a patient care team prior to surgical treatment, and promoting a safe postoperative period.

## 1. Introduction

The complications of thoracic aortic disease include aortic dissection and aneurysm. These pathologies are common for elderly patients, males, smokers, and those with a family history of aneurysms. More than 20% of patients with aortic disease, suffering from acute aortic events, have no symptoms and die at home, without receiving medical help [1].

The causes of death include not only aortic rupture, but also myocardial infarction, renal insufficiency, and stroke [2]. In combination with several cardiovascular comorbidities, these factors complicate clinical decision making. One of the ways to decrease a patient’s risk is to ensure a timely prognosis of complications. 

Despite the fact that various risk scales (Euroscore, Euroscore II, STS score) are successfully used in cardiac surgery, there is still no single prognostic risk assessment scale for patients with thoracic aortic pathology. Currently, there are several attempts being made to design specific predictive models for thoracic aortic pathology risk assessment [3,4]. However, extension of the dataset is required to identify the most significant risk factors, due to the heterogeneity in the obtained predictors in all studies. The significant risk factors are used to create a scale that is correct for assessing perioperative risk in patients with thoracic aorta. 

Machine learning (ML) can provide tools for personalized risk prediction based on real-world data and the clinical history of a patient [5]. It employs collected routine clinical data to implement mathematical models that can forecast risks [6]. The ML models can predict the expansion of aortic aneurysm based on the anatomical features extracted from CT scans and textual documents. The ML algorithm developed by Hirata et al. [7] could predict an expansion of an aneurysm with high accuracy. Another study used ML techniques to make a prognosis on the risk of aortic aneurysm growth in 85% and 71% of patients at 12 and 24 months, respectively [8].

The incidence of adverse events is not the same in each patient. The evaluation of risk factors for adverse events in patients after such a complex procedure is crucial. To date, some authors have attempted to identify predictors of early postoperative complications [4,9,10,11]. However, searching the predictors for perioperative and postoperative complications and mortality after thoracic aortic surgery is still an issue. Recent studies have investigated the problem of TAA and related risks. 

Table 1 summarizes the results of the review performed for cardiovascular predictive modelling.

The algorithms most frequently used for cardiovascular predictive modelling are logistic regression (LR), ensemble models and tree models (random forest and decision tree classifiers), and boosting strategies, such as XGBoost. The most frequent metric for the evaluation of predictive models is the area under the receiver operating characteristic curve (AUC-ROC). Thereby, a higher value corresponds to better discrimination [17].

The goal of the presented study is to develop predictive models for significant risk factor identification in patients after thoracic aneurysm surgeries, using integrated data from different medical institutions.

## 2. Materials and Methods

The model for risk prognosis was developed using two datasets from two clinical providers. The first dataset contains 97 structured records for 137 patients with clinical records on aortic operations. The second dataset contains 56,929 text documents from the years 2008–2019 for the 343 TAA operations of 319 patients. 

We formulated seven target features: in-hospital mortality; temporary neurological deficit (TND); permanent neurological deficit (PND); prolonged (>7 days) lung ventilation (LV); renal replacement therapy (RRT); myocardial infarction (MI); multiple organ failure (MOF). In total, 61 input parameters were used for the risk prediction model. The features were organized in the following categories: anthropometric data (6 features), comorbidities (8 features), laboratory tests (5 features), coronary angiographic data (4 features), echocardiographic data (8 features), computed tomographic data (14 features), intraoperative data (15 features), and concomitant cardiac procedures (3 features). The full feature list is available in Appendix A.

**Figure 1:**
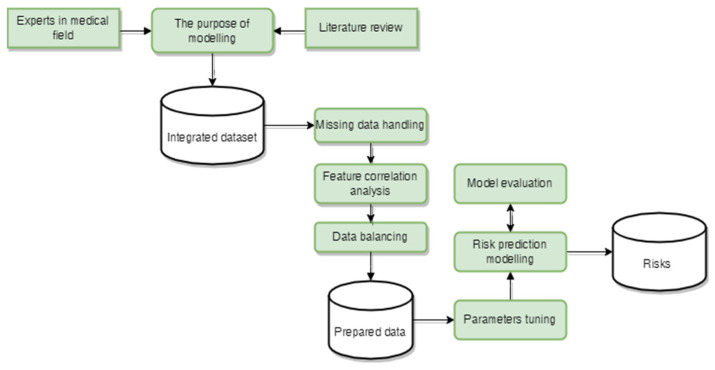
The pipeline for medical risk model development.

The pipeline for the model development is represented in Figure 1.

The features in the dataset with >30% missing values were eliminated. For managing features with up to 30% missing values, the k nearest neighbors (KNN) imputation technique was applied. The Pearson’s correlation method was used for feature correlation analysis. Features with a high correlation coefficient were eliminated. The synthetic minority over-sampling technique (SMOTE) was employed for balancing the dataset. The classification was conducted using the two most important features, and all of the features were used to compare performances. The feature selection was organized through the voting of several techniques: univariate feature selection with a chi-squared test, recursive feature elimination (RFE), extra trees classifier, and Lasso.

We used logistic regression (LR), random forest (RF) and CatBoost (CC) classifiers for experiments. The parameters were tuned through the grid search, and the F-score was used as the optimization metric. 

LR is expressed by the following equation:

(1) $$Z=\frac{1}{1+e^{-\left(\beta_{0}+\beta_{1}x\right)}}$$

LR is the most frequently used machine learning model in medical applications, due to its high interpretability. Its sensitivity to the multicollinearity problem is one of the disadvantages of the LR model. Thus, highly correlated features should not be included in the predictive model. 

RF is an ensemble model based on decision trees. During classification, each tree assigns the most likely target to each patient with a set of predictors. The averaging function is expressed by the following equation:

(2) $$Z=argmax\frac{1}{T}\overset{T}{\underset{t=1}{\sum }}p_{t}(y|x)$$

where *p_t_* (*y*|*x*) is the probability distribution for each tree. RF is also a widespread algorithm for medical applications. 

CatBoost is an ordered gradient boosting algorithm that addresses the problem of target leakage. CC is effective on small datasets. Binary decision trees are used in the CC classifier. The CC output can be expressed as follows:

(3) $$Z=H\left(x_{i}\right)=\overset{J}{\underset{j=1}{\sum }}c_{j}1_{\left\{x\in R_{j}\right\}}$$

*H*(*x_i_*) is a decision tree function and *R_j_* is a disjoint region corresponding to the leaves of the tree.

The experiments were conducted with the following Python 3 packages: scikit-learn [18] and CatBoost [19] for machine learning model implementation; seaborn [20] and matplotlib [21] for data visualization; SMOTE [22] for dataset balancing; and SHapley Additive exPlanations (SHAP) [23] for the interpretation of black-box results. The discrimination was evaluated using ROC curves. 

Table 2 lists the machine learning models and parameters used in the research.

## 3. Results

Table 3 shows the best performances for each classification target.

**Figure 2:**
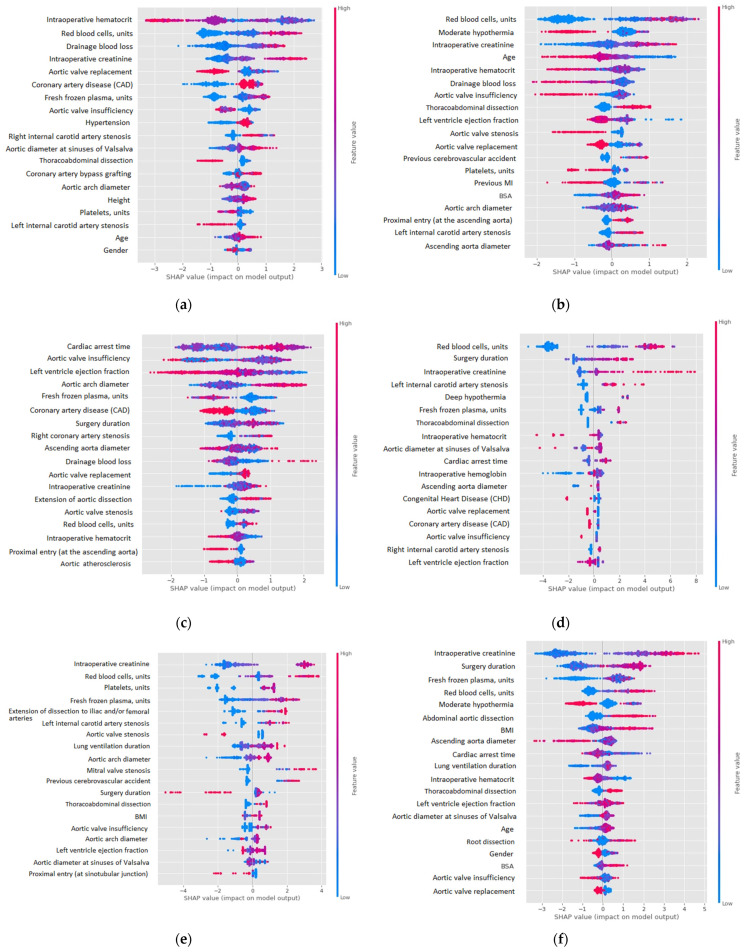

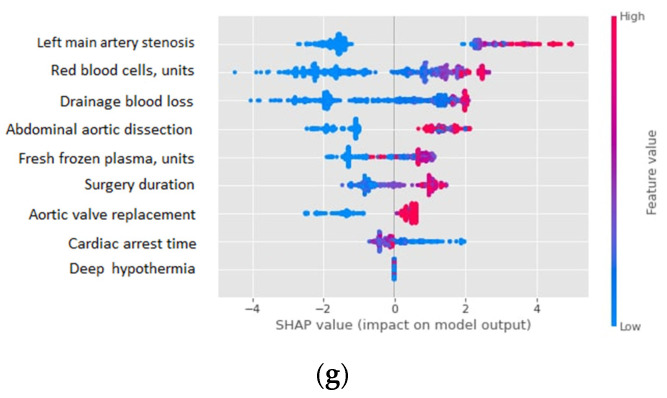
Feature importance diagrams for target variables: (**a**) in-hospital mortality; (**b**) TND; (**c**) PND; (**d**) prolonged lung ventilation; (**e**) RRT; (**f**) MOF; (**g**) MI.

Figure 2 represents the interpretation of the CatBoost classifier results for each target variable. The diagram shows the impact of each feature on the model output.

The red color in Figure 2 relates to a higher value of the feature (for binary features, it corresponds to one), while the blue color corresponds to a lower feature value. The negative SHAP value corresponds to a negative impact on prediction, leading the model to predict zero, and a positive SHAP value corresponds to a positive impact on prediction, leading the model to predict one. For instance, a higher intraoperative hematocrit leads to a lower mortality risk, and a lower intraoperative hematocrit leads to a higher mortality risk. A decreased level of red blood cells leads to lower risks of TND cases, but a decreased level of red blood cells does not necessarily lead to higher risks of TND cases. 

**Figure 3:**
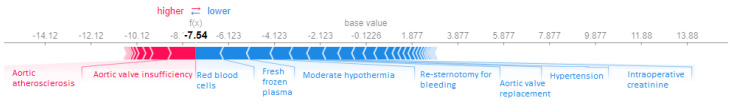
The example of a single patient’s prediction.

Figure 3 represents the plot, showing the most powerful predictors for a particular patient from the dataset for in-hospital mortality.

The bold value in Figure 3 indicates the model’s output value. The red features increase the prediction and the blue features decrease the prediction. Aortic valve insufficiency has a positive impact on the output value and the red blood cell feature has a negative impact on the output value.

## 4. Discussion

Despite the fact that a number of scoring systems for cardiac risk assessment have been developed and successfully applied in practice, they do not take into account the specificity of thoracic aortic pathology. More and more medicine-related studies concentrate on building machine learning models to learn from historical experience [24], and to identify specific risk factors.

Currently, there are a number of studies devoted to the identification of prognostic factors for postoperative outcomes in patients with thoracic aortic pathology. Age, NYHA III–IV class of heart failure, renal insufficiency, ascending aorta dilatation, involvement of the aortic arch in the pathological process, lower limb malperfusion, and emergent/urgent aortic surgery are the most common risk factors that affect the survival and development of postoperative complications. In addition, the likelihood of a favorable prognosis decreases, due to reoperations, combined cardiac surgery (e.g., coronary artery bypass grafting), and a prolonged cardiopulmonary bypass duration [4,11]. Some studies have emphasized the negative role of increased blood components in transfusions (packed red blood cells, fresh frozen plasma, and platelets) [4,9,10]. 

Great attention is paid to the prognostic criteria for thoracic aortic surgery; however, there are few studies that aim to identify the relationship between risk factors and adverse outcomes. This study is dedicated to the development of a predictive model based on integrated medical data, using two datasets from high-throughput aortic centers. 

Feature selection plays an important role in medical risk prediction using machine learning models. We removed six features due to discrepancies in the data storage formats and in the diagnostic methods applied in the participating clinics, and because of the missing values. The exploratory data analysis resulted in the removal of weight, due to the high correlation with two other features. The circulatory arrest time, cardioplegic arrest time, and cardiopulmonary bypass time were eliminated because of the large number of missing values, as shown in [25], acknowledging that the application of imputation methods can distinctly affect the performance of the predictive model. 

We tested three machine learning algorithms to develop a predictive model: (1) LR; (2) RF; (3) CatBoost. CatBoost, with the SMOTE balancing technique, demonstrated the best performance for the most targets. 

We demonstrated several tools for CatBoost evaluation and interpretation: featuring importance scores, which are summarized using summary plots for each target variable (Figure 2); comparison with other well-known machine learning models (LR and RF), using metrics such as ROC AUC, F-score, Recall, and Precision (Table 3). An accuracy measurement can be misleading, due to the fact that higher metric values indicate overfitting, especially on imbalanced datasets [26]. Precision is the ratio between correctly classified patients and all patients assigned to the class. Recall is the rate of correctly classified patients. If recall equals one, the prediction of positive classes is perfect. This metric is crucial to evaluate medical prediction models, as it is important to identify as many cases of the pathological event as possible. A low recall value corresponds to a high rate of positive cases of medical risk missed. F-score is the harmonic mean of recall and precision. The use of F-score in parameter tuning helps to penalize models for extreme values [27]. 

The SHAP value was used to ensure interpretability of the model. SHAP covers two aspects: global and local interpretability. Global interpretability explains the relationships of predictors with target variables, i.e., risk factors with risks, and allows the consistency of the model to be analyzed with the current practices. Local interpretability helps to understand why a particular case or patient obtains a particular prediction.

Figure 2 illustrates the summary plots for each target variable, showing negative and positive relationships of predictors with targets. These plots take into account the feature importance, the impact of each feature on the final prediction, the initial value of the feature (lower values are blue and higher values are red), and the correlation of the feature with the target (lower intraoperative creatinine correlates with a lower risk of multiple organ failure). The SHAP value provides the correlation, but not causation.

Figure 3 illustrates an example of a force plot for a single patient from the dataset. It helps to understand the influence of each predictor on the final output. Such a plot might be useful for future decision making.

The performance of the developed models could be compared to the results of other studies in predicting postoperative cardiovascular complications. Coulson et al. [16] set an aim to develop models to predict the risks of acute kidney injury and the need for renal replacement therapy after cardiac surgery, using as few predictors as possible. The simplicity and interpretability of the models, and the few predictors used, ensure the accessability of prediction models for clinicians. Thus, a careful analysis of the literature and accumulated practical experience is needed to stratify risk factors. The AUC ROC for the acute kidney injury postoperative prediction was 0.70, and the AUC ROC for the need for renal replacement therapy postoperative prediction was 0.85. 

Fernandes et al. [15] investigated machine learning models to predict mortality after cardiac surgery. The best results were shown by boosting classifiers and random forest, showing 0.87 AUC ROC and up to 0.91 recall. 

Czerny et al. [3] showed that logistic regression outperformed the other investigated classifiers, with a mean AUC of 0.712 for predicting mortality rate in acute aortic dissection.

The CatBoost classifier performs better in comparison with the results from the literature.

In most cases, the obtained results are in line with the current literature. Thus, the independent risk factors for postoperative acute kidney injury requiring RRT are impaired preoperative renal function, reduced left ventricle ejection fraction, and transfusion of a large volume of blood components, as well as being overweight [28,29,30]. In our model, these factors contribute significantly to the postoperative acute kidney injury.

Additionally, Wang et al. [11] demonstrated that the large extent of aortic dissection was an independent risk factor for early mortality. In another study, a significant negative role of primary fenestration with aortic dissection, especially with type B, was revealed as an important factor for mortality [31]. Moreover, the presence of this type of aortic dissection led to an increase in postoperative renal complications [32]. In another study, an enlarged abdominal aortic diameter was shown to be a risk factor for complications in the postoperative period [33].

Nevertheless, we should point out that, from a clinical perspective, the impact of many features in the predictive model is obscure. However, most of the features have a logical clinical explanation. The example of such clinical significance is a direct relation of the aortic diameter at the sinuses of Valsalva to temporal neurological deficit, which is still indistinct. To reveal the answer, one needs to resolve a logical chain. A large aortic root is an indication that it has been replaced. This naturally prolongs the cardiopulmonary bypass time and, successively, increases the risk of neurological deficiency. 

Despite the successful implementation of surgical risk calculators (Euroscore, Euroscore II, and STS score), a standardized prognostic risk assessment scale for patients with thoracic aortic pathology has not yet been adopted. In the current literature, there have been a few attempts to compile prognostic models [4]. However, due to the heterogeneity of the predictors obtained in each particular study, the accumulation of more data is needed, in order to identify the significant risk factors. Elaboration of the correct risk score calculation for prognosis assessment in patients with thoracic aortic diseases is crucial. Our findings provide additional support for clinical decision making, guiding a patient care team prior to a surgical treatment, and promoting a safe postoperative period.

The presented study has certain limitations. Despite the integration of medical records from the datasets of two different clinics, the number of patients and clinical cases (operations) is relatively small. We are planning to extend it in the future. The study faced a problem of unbalanced data, which is a traditional concern for medical data [12]. This leads to situations where machine learning algorithms tend to classify the data into predominant classes. SMOTE for data balancing, and F-measure as the optimization metric, which is less sensitive to data imbalance, were applied to address the problem. However, the study still has limitations due to the imbalanced medical datasets. Another limitation is related to the loss of data during the integration process. We had to compare and map not only the logical data structures and contents, but also diagnostic methods and treatment approaches in different institutions. This reduced the amount of data we could include in the study. 

## 5. Conclusions

This study has implemented models for postoperative risk prognosis for patients with thoracic aortic disease, using real-world data from two different medical institutions, comprising from both structured data and free-text medical records. The obtained findings provide additional support for clinical decision making, guiding a patient care team prior to surgical treatment, and promoting a safe postoperative period. Future studies may address the current limitations of the study, such as relevant synthetic patients’ generation, model validation in a medical practice, and the development of applied risk stratification scales based on the obtained results.

## Figures and Tables

**Table 1:** Recent studies for cardiovascular predictive modelling.

Scheme	Algorithm	AUC-ROC	Data	Target
Lee, 2018 [12]	XGBoost	0.78	Open heart and TAA surgery	Acute kidney injury
Zhong, 2021 [13]	XGBoost	0.93	Coronary artery bypass surgery, aortic valve replacement and other heart surgeries	30-day mortality, septic shock, liver dysfunction, and thrombocytopenia
Allyn, 2017 [14]	Model ensemble	0.78	Elective heart surgery	Postoperative mortality
Fernandes, 2021 [15]	XGBoost	0.88	Intraoperative open heart surgery data	Postoperative mortality
Coulson, 2020 [16]	Logistic regression	0.78–0.85	Open heart surgery	Acute kidney injury

**Table 2:** Models and parameters.

Model	Parameters
LR* (imp. feat.)	‘C’: 2.83, ‘solver’: ‘newton-cg’
LR + SMOTE (imp. feat.)	‘C’: 0.5, ‘solver’: ‘newton-cg’
LR + SMOTE (all feat.)	‘C’: 4.0, ‘solver’: ‘liblinear’
RF (imp. feat.)	‘criterion’: ‘gini’, ‘max_features’: ‘auto’
RF + SMOTE (imp. feat.)	‘criterion’: ‘gini’, ‘max_features’: ‘auto’
RF + SMOTE (all feat.)	‘criterion’: ‘gini’, ‘max_features’: ‘log2’
CC * (all. feat.)	‘depth’: 4, ‘l2_leaf_reg’: 3, ‘learning_rate’: 0.6
CC + SMOTE (imp. feat.)	‘depth’: 5, ‘l2_leaf_reg’: 2, ‘learning_rate’: 0.9
CC + SMOTE (all feat.)	‘depth’: 4, ‘l2_leaf_reg’: 1, ‘learning_rate’: 0.2

* LR–logistic regression, RF—random forest, CC—CatBoost classifier; imp. feat.—the model is composed using only important features, all feat.—the model is composed using all available features.

**Table 3:** Performance of the classifiers for each target.

Target	Best Classifier	ROC AUC	F-Score	Recall	Precision
In-hospital mortality	CC * + SMOTE (all feat.)	0.965	0.966	0.992	0.942
Temporary neurological deficit (TND)	CC + SMOTE (all feat.)	0.960	0.959	0.936	0.983
Permanent neurological deficit (PND)	CC + SMOTE (all feat.)	0.946	0.947	0.969	0.926
Prolonged lung ventilation (>7 days)	CC + SMOTE (all feat.)	0.957	0.958	0.984	0.934
Renal replacement therapy (RRT)	CC + SMOTE (all feat.)	0.985	0.984	0.992	0.978
Myocardial infarction (MI)	CC + SMOTE (imp. feat.)	0.986	0.984	0.993	0.979
Multiple organ failure (MOF)	CC + SMOTE (all feat.)	0.952	0.950	0.964	0.958

* CC—CatBoost classifier; imp. feat.—the model is composed using only important features, all feat.—the model is composed using all available features.

## Data Availability

Not applicable.

## Appendix A

Endpoints:

1. In-hospital mortality;
2. Temporary neurological deficit (TND);
3. Permanent neurological deficit (PND);
4. Prolonged lung ventilation (LV) (>7 days);
5. Renal replacement therapy (RRT);
6. Myocardial infarction (MI);
7. Multiple organ failure (MOF).

Anthropometric data:

1. Gender;
2. Age;
3. Height;
4. Weight;
5. Body mass index (BMI);
6. Body surface area (BSA).

Comorbidities:

1. Congenital heart disease (CHD);
2. Hypertension;
3. Coronary artery disease (CAD);
4. Previous MI;
5. Previous cerebrovascular accident;
6. Chronic obstructive pulmonary disease (COPD);
7. Marfan syndrome;
8. Aortic atherosclerosis.

Laboratory tests:

1. Preoperative hematocrit;
2. Preoperative urea;
3. Preoperative creatinine;
4. Preoperative glomerular filtration rate (GFR).

Coronary angiographic data:

1. Left main artery stenosis (LMA);
2. Right coronary artery stenosis (RCA);
3. Obtuse margin artery stenosis (OMA);
4. Left anterior descending artery (LAD).

Echocardiographic data:

1. Left internal carotid artery stenosis;
2. Right internal carotid artery stenosis;
3. Left ventricle ejection fraction;
4. Aortic valve stenosis;
5. Aortic valve insufficiency;
6. Mitral valve stenosis;
7. Mitral valve insufficiency;
8. Aortic diameter at sinuses of Valsalva.

Computed tomographic data:

1. Ascending aorta diameter;
2. Aortic arch diameter;
3. Segment A diameter = proximal descending aortic diameter;
4. Segment B diameter = distal descending aortic diameter;
5. Segment C diameter = abdominal aortic diameter;
6. Proximal entry (at sinotubular junction);
7. Proximal entry (at the ascending aorta);
8. Proximal entry (at the aortic arch);
9. Proximal entry behind the left subclavian artery = type B aortic dissection;
10. Involvement aortic root in dissection;
11. Involvement ascending aortic in dissection;
12. Involvement aortic arch in dissection;
13. Thoracoabdominal dissection;
14. Abdominal aortic dissection;
15. Extension of aortic dissection down to iliac and/or femoral arteries.

Intraoperative data:

1. Cardiac arrest time;
2. Antegrade cerebral perfusion time;
3. Circulatory arrest time;
4. Deep hypothermia;
5. Moderate hypothermia;
6. Re-sternotomy for bleeding;
7. Surgery duration;
8. Red blood cells, units;
9. Fresh frozen plasma, units;
10. Platelets, units;
11. Drainage blood loss;
12. Intraoperative hematocrit.
13. Intraoperative creatinine.

Concomitant cardiac procedures:

1. Coronary artery bypass grafting;
2. Aortic valve replacement;
3. Mitral valve replacement.
